# Supplementary material for: Machine learning-based prediction models unleash the enhanced production of fucoxanthin in Isochrysis galbana
Source: Front Plant Sci. 2024 Oct 16;15:1461610. doi: 10.3389/fpls.2024.1461610 (PMC11521944; doi:10.3389/fpls.2024.1461610)
Supplement: Supplementary Figure 1 — Schematic representation of Artificial neural network ML model. The input layer comprises four neurons corresponding to the four input variables (type of hormone, concentration of hormones, growth rate and biomass) and the output layer has one neuron corresponding to fucoxanthin yield. Three hidden layers with 4, 3, and 4 neurons respectively were found to give good performance. The tan h activation function was utilized in this study for the computation of output. The regularization value of α=0.0006 and L-BFGS-B solver was used for the ANN model. The neurons between adjacent layers are fully interconnected and the number of iterations was set to 200 for the training algorithm to reduce the error between the actual and predicted output. [file DataSheet1.docx]

**Supplementary Table 1. Concentrations of phytohormones used for culturing *Isochrysis galbana*.**

| **Phytohormone supplementation** | **Notation used for ML model** | **Abbreviation** | **Concentration (mg l^-1^)** |
| --- | --- | --- | --- |
| Control | C | - | - |
| Indoleacetic acid (IAA) | I 1 | IAA (0.02) | 0.02 |
|  |  | IAA (0.2) | 0.2 |
|  |  | IAA (2) | 2 |
|  |  | IAA (20) | 20 |
| Salicylic acid (SA) | I 2 | SA (0.02) | 0.02 |
|  |  | SA (0.2) | 0.2 |
|  |  | SA (2) | 2 |
|  |  | SA (20) | 20 |
| Gibberellic acid (GA_3_) | I 3 | GA_3_ (0.02) | 0.02 |
|  |  | GA_3_ (0.2) | 0.2 |
|  |  | GA_3_ (2) | 2 |
|  |  | GA_3_ (20) | 20 |
| Methyl jasmonate (MeJa) | I 4 | MeJa (0.02) | 0.02 |
|  |  | MeJa (0.2) | 0.2 |
|  |  | MeJa (2) | 2 |
|  |  | MeJa (20) | 20 |

**Supplementary Table 2. Maximum growth rate and fucoxanthin yield attained for various phytohormones.**

| **Phytohormones** | **Concentration (mg l^-1^)** | **Days of maximum yield** | **Maximum growth rate** | **Maximum fucoxanthin yield (μg ml^-1^)** |
| --- | --- | --- | --- | --- |
| Control | 0 | Day 18-20 | 0.738 | 4.6 |
| I1 | 0.02 | Day 10-15 | 0.827 | 5.1 |
|  | 0.2,2,20 | Day 15-20 | 0.96 | 5.6 |
| I2 | 0.02 | Day 10-15 | 0.78 | 4.69 |
|  | 0.2 | Day 15-20 | 0.85 | 5.2 |
| I3 | 0.02. 0.2 | Day 16 | 0.9 | 4.9 |
|  | 2 | Day 14 | 0.85 | 4.1 |
| I4 | 0.02 | Day 14 | 0.903 | 5.02 |
|  | 0.2 | Day <10 | 0.812 | 7.83 |

**Supplementary Table 3. Prediction of fucoxanthin yield by ML models trained with all input variables excluding hormone descriptors**

| **S. No.** | **Name of the phytohormone** | **Concentration (mg l^-1^)** | **Days** | **Growth rate** | **Actual measured fucoxanthin yield (μg ml^-1^)** | **Predicted fucoxanthin yield by ML models** | | | |
| --- | --- | --- | --- | --- | --- | --- | --- | --- | --- |
|  |  |  |  |  |  | **RF** | **LR** | **SVM** | **ANN** |
| 1 | Control | 0 | 16 | 0.73 | 2.699 | 3.356 | 2.397 | 2.451 | 1.94 |
| 2 | Methyl jasmonate | 0.02 | 8 | 0.678 | 0.933 | 1.899 | 2.182 | 1.869 | 0.918 |
| 3 | Indole acetic acid | 2 | 10 | 0.757 | 3.769 | 2.688 | 2.538 | 2.482 | 3.321 |
| 4 | Indole acetic acid | 2 | 18 | 0.969 | 4.276 | 4.592 | 3.832 | 4.132 | 4.552 |
| 5 | Gibberellic acid | 0.2 | 14 | 0.899 | 4.923 | 4.415 | 3.419 | 3.364 | 4.262 |
| 6 | Salicylic acid | 0.02 | 12 | 0.717 | 2.633 | 3.099 | 1.976 | 1.865 | 2.06 |
| 7 | Methyl jasmonate | 0.2 | 14 | 0.963 | 5.35 | 4.542 | 3.89 | 4.022 | 4.521 |
| 8 | Gibberellic acid | 0.02 | 24 | 0.746 | 0.107 | 1.306 | 2.599 | 1.752 | 0.561 |
| 9 | Gibberellic acid | 0.2 | 10 | 0.791 | 3.413 | 3.241 | 2.761 | 2.461 | 3.031 |
| 10 | Salicylic acid | 0.2 | 16 | 0.811 | 4.82 | 4.081 | 2.553 | 2.656 | 4.656 |
| 11 | Abscisic acid | 0.02 | 10 | 0.725 | 0 | 3.289 | 2.326 | 2.42 | 2.779 |
| 12 | Abscisic acid | 0.02 | 14 | 0.624 | 0.4 | 0.411 | 1.763 | 1.344 | 1.479 |
| 13 | Abscisic acid | 0.02 | 6 | 0.649 | 0 | 0.998 | 1.857 | 1.533 | 2.18 |
| 14 | Abscisic acid | 0.2 | 8 | 0.667 | 0 | 2.042 | 1.976 | 1.761 | 2.217 |
| 15 | Abscisic acid | 0.2 | 10 | 0.709 | 0 | 2.809 | 2.234 | 2.245 | 2.557 |
| 16 | Abscisic acid | 0.2 | 12 | 0.641 | 0.6 | 0.84 | 1.849 | 1.532 | 1.659 |
| 17 | Abscisic acid | 2 | 10 | 0.689 | 0 | 1.531 | 2.122 | 1.991 | 2.674 |
| 18 | Abscisic acid | 2 | 14 | 0.463 | 0 | 0.099 | 0.828 | -0.098 | 0.491 |

**Supplementary Table 4. Test results of ML models trained with all input variables including hormone descriptors.**

| **Model** | **MSE** | **RMSE** | **MAE** | ***R*^2^** |
| --- | --- | --- | --- | --- |
| Random Forest | 0.552 | 0.743 | 0.42 | 0.825 |
| Linear Regression | 1.25 | 1.118 | 0.907 | 0.604 |
| Support Vector Machine | 1.468 | 1.212 | 0.815 | 0.535 |
| Artificial Neural Network | 0.801 | 0.895 | 0.497 | 0.746 |

**Supplementary Table 5. Prediction of fucoxanthin yield by ML models trained with all input variables.**

| **S. No.** | **Name of the phytohormone** | **Concentration (mg l^-1^)** | **Days** | **Growth rate** | **Actual measured fucoxanthin yield (μg ml^-1^)** | **Predicted fucoxanthin yield by ML models** | | | |
| --- | --- | --- | --- | --- | --- | --- | --- | --- | --- |
|  |  |  |  |  |  | **RF** | **LR** | **SVM** | **ANN** |
| 1 | Control | 0 | 16 | 0.73 | 2.699 | 3.408 | 2.85E+08 | 1.409 | 3.49 |
| 2 | Methyl jasmonate | 0.02 | 8 | 0.678 | 0.933 | 1.682 | -2.02E+08 | 1.609 | 1.803 |
| 3 | Indole acetic acid | 2 | 10 | 0.757 | 3.769 | 3.059 | 6.22E+07 | 2.082 | 3.124 |
| 4 | Indole acetic acid | 2 | 18 | 0.969 | 4.276 | 5.07 | 6.22E+07 | 3.169 | 5.198 |
| 5 | Gibberellic acid | 0.2 | 14 | 0.899 | 4.923 | 4.767 | 6.22E+07 | 2.472 | 4.594 |
| 6 | Salicylic acid | 0.02 | 12 | 0.717 | 2.633 | 3.229 | 6.22E+07 | 1.619 | 2.388 |
| 7 | Methyl jasmonate | 0.2 | 14 | 0.963 | 5.35 | 5.121 | -2.02E+08 | 2.813 | 5.598 |
| 8 | Gibberellic acid | 0.02 | 24 | 0.746 | 0.107 | 1.34 | 6.22E+07 | 1.841 | 0.504 |
| 9 | Gibberellic acid | 0.2 | 10 | 0.791 | 3.413 | 3.592 | 6.22E+07 | 2.023 | 3.12 |
| 10 | Salicylic acid | 0.2 | 16 | 0.811 | 4.82 | 4.373 | 6.22E+07 | 2.064 | 4.568 |
| 11 | Abscisic acid | 0.02 | 10 | 0.725 | 0 | 2.945 | -7.59E+09 | 1.94 | 5.012 |
| 12 | Abscisic acid | 0.02 | 14 | 0.624 | 0.4 | 0.695 | -7.59E+09 | 1.397 | 2.239 |
| 13 | Abscisic acid | 0.02 | 6 | 0.649 | 0 | 0.614 | -7.59E+09 | 1.507 | 3.644 |
| 14 | Abscisic acid | 0.2 | 8 | 0.667 | 0 | 1.959 | -7.59E+09 | 1.614 | 3.972 |
| 15 | Abscisic acid | 0.2 | 10 | 0.709 | 0 | 2.394 | -7.59E+09 | 1.851 | 4.744 |
| 16 | Abscisic acid | 0.2 | 12 | 0.641 | 0.6 | 0.707 | -7.59E+09 | 1.486 | 2.83 |
| 17 | Abscisic acid | 2 | 10 | 0.689 | 0 | 1.964 | -7.59E+09 | 1.717 | 4.191 |
| 18 | Abscisic acid | 2 | 14 | 0.463 | 0 | 0 | -7.59E+09 | 0.561 | 0.163 |

**Supplementary Table 6. Prediction of growth rate by ML models trained with restricted pre-processed input data**

| **S. No.** | **Name of the phytohormone** | **Concentration (mg l^-1^)** | **Days** | **Actual measured growth rate** | **Predicted growth rate using ML models** | | | |
| --- | --- | --- | --- | --- | --- | --- | --- | --- |
|  |  |  |  |  | **RF** | **LR** | **SVM** | **ANN** |
| 1 | Control | 0 | 16 | 0.73 | 0.728 | 0.577 | 0.613 | 0.737 |
| 2 | Methyl jasmonate | 0.02 | 8 | 0.678 | 0.737 | 0.526 | 0.62 | 0.739 |
| 3 | Indole acetic acid | 2 | 10 | 0.757 | 0.715 | 0.644 | 0.739 | 0.574 |
| 4 | Indole acetic acid | 2 | 18 | 0.969 | 0.87 | 0.611 | 0.811 | 0.745 |
| 5 | Gibberellic acid | 0.2 | 14 | 0.899 | 0.862 | 0.549 | 0.726 | 0.833 |
| 6 | Salicylic acid | 0.02 | 12 | 0.717 | 0.67 | 0.628 | 0.709 | 0.649 |
| 7 | Methyl jasmonate | 0.2 | 14 | 0.963 | 0.832 | 0.5 | 0.687 | 1.249 |
| 8 | Gibberellic acid | 0.02 | 24 | 0.746 | 0.709 | 0.51 | 0.611 | 0.752 |
| 9 | Gibberellic acid | 0.2 | 10 | 0.791 | 0.752 | 0.565 | 0.693 | 0.632 |
| 10 | Salicylic acid | 0.2 | 16 | 0.811 | 0.786 | 0.611 | 0.75 | 0.736 |
| 11 | Abscisic acid | 0.02 | 10 | 0.403 | 0.701 | 0.606 | 0.687 | 0.133 |
| 12 | Abscisic acid | 0.02 | 14 | 0.682 | 0.852 | 0.59 | 0.732 | 0.206 |
| 13 | Abscisic acid | 0.02 | 6 | 0.28 | 0.656 | 0.623 | 0.592 | 0.159 |
| 14 | Abscisic acid | 0.2 | 8 | 0.348 | 0.691 | 0.613 | 0.644 | 0.145 |
| 15 | Abscisic acid | 0.2 | 10 | 0.561 | 0.711 | 0.605 | 0.686 | 0.132 |
| 16 | Abscisic acid | 0.2 | 12 | 0.551 | 0.815 | 0.597 | 0.715 | 0.148 |
| 17 | Abscisic acid | 2 | 10 | 0.48 | 0.658 | 0.59 | 0.675 | 0.129 |
| 18 | Abscisic acid | 2 | 14 | 0.521 | 0.774 | 0.573 | 0.721 | 0.427 |
| 19 | Abscisic acid | 20 | 10 | 0.51 | 0.441 | 0.438 | 0.51 | 0.141 |

**Supplementary Table 7. Prediction of fucoxanthin yield by generic ML models trained with restricted pre-processed input data**

| **S. No.** | **Name of the phytohormone** | **Concentration (mg l^-1^)** | **Days** | **Growth rate** | **Actual measured fucoxanthin yield (μg ml^-1^)** | **Predicted fucoxanthin yield by ML models** | | | |
| --- | --- | --- | --- | --- | --- | --- | --- | --- | --- |
|  |  |  |  |  |  |  |  |  |  |
|  |  |  |  |  |  | **RF** | **LR** | **SVM** | **ANN** |
| 1 | Control | 0 | 16 | 0.73 | 2.699 | 3.112 | 2.68 | 1.722 | 3.117 |
| 2 | Methyl jasmonate | 0.02 | 8 | 0.678 | 0.933 | 0.751 | 2.244 | 1.438 | 0.052 |
| 3 | Indole acetic acid | 2 | 10 | 0.757 | 3.769 | 2.881 | 2.558 | 2.232 | 3.118 |
| 4 | Indole acetic acid | 2 | 18 | 0.969 | 4.276 | 4.691 | 3.767 | 3.482 | 4.993 |
| 5 | Gibberellic acid | 0.2 | 14 | 0.899 | 4.923 | 4.817 | 3.354 | 2.112 | 4.861 |
| 6 | Salicylic acid | 0.02 | 12 | 0.717 | 2.63 | 2.92 | 1.963 | 1.834 | 2.99 |
| 7 | Methyl jasmonate | 0.2 | 14 | 0.963 | 5.35 | 5.149 | 3.872 | 2.908 | 5.611 |
| 8 | Gibberellic acid | 0.02 | 24 | 0.746 | 0.107 | 0.894 | 2.447 | 1.138 | 0.36 |
| 9 | Gibberellic acid | 0.2 | 10 | 0.791 | 3.413 | 3.253 | 2.738 | 1.597 | 3.136 |
| 10 | Salicylic acid | 0.2 | 16 | 0.811 | 4.82 | 5.186 | 2.499 | 2.32 | 4.398 |
| 11 | Abscisic acid | 0.02 | 10 | 0.725 | 0 | 2.946 | 2.275 | 1.712 | -0.109 |
| 12 | Abscisic acid | 0.02 | 14 | 0.624 | 0.4 | 0.279 | 1.696 | 1.051 | 0.015 |
| 13 | Abscisic acid | 0.02 | 6 | 0.649 | 0 | 0.762 | 1.843 | 1.278 | -0.054 |
| 14 | Abscisic acid | 0.2 | 8 | 0.667 | 0 | 0.913 | 1.946 | 1.37 | -0.076 |
| 15 | Abscisic acid | 0.2 | 10 | 0.709 | 0 | 1.93 | 2.185 | 1.612 | -0.106 |
| 16 | Abscisic acid | 0.2 | 12 | 0.641 | 0.6 | 0.713 | 1.795 | 1.175 | -0.031 |
| 17 | Abscisic acid | 2 | 10 | 0.689 | 0 | 1.963 | 2.079 | 1.466 | -0.086 |
| 18 | Abscisic acid | 2 | 14 | 0.463 | 0 | 0 | 0.787 | 0.167 | 0.026 |

**Supplementary Table 8. Test results of growth rate prediction by ML models trained with raw data.**

| **Model** | **MSE** | **RMSE** | **MAE** | ***R*^2^** |
| --- | --- | --- | --- | --- |
| Random Forest | 0.012 | 0.11 | 0.082 | 0.803 |
| Linear Regression | 0.052 | 0.228 | 0.195 | 0.156 |
| Support Vector Machine | 0.028 | 0.166 | 0.135 | 0.551 |
| Artificial Neural Network | 0.009 | 0.097 | 0.076 | 0.846 |

**Supplementary Table 9. Prediction of growth rate using raw data**

| **S. No.** | **Name of the phytohormone** | **Concentration (mg l^-1^)** | **Days** | **Actual measured growth rate** | **Predicted growth rate using ML models** | | | |
| --- | --- | --- | --- | --- | --- | --- | --- | --- |
|  |  |  |  |  | **RF** | **LR** | **SVM** | **ANN** |
| 1 | Control | 0 | 16 | 0.73 | 0.779 | 0.585 | 0.277 | 1.697 |
| 2 | Methyl jasmonate | 0.02 | 8 | 0.678 | 0.642 | 0.527 | 0.644 | 0.565 |
| 3 | Indole acetic acid | 2 | 10 | 0.757 | 0.759 | 0.675 | 0.703 | 0.574 |
| 4 | Indole acetic acid | 2 | 18 | 0.969 | 0.92 | 0.637 | 0.748 | 0.897 |
| 5 | Gibberellic acid | 0.2 | 14 | 0.899 | 0.875 | 0.549 | 0.726 | 0.998 |
| 6 | Salicylic acid | 0.02 | 12 | 0.717 | 0.765 | 0.619 | 0.698 | 0.679 |
| 7 | Methyl jasmonate | 0.2 | 14 | 0.963 | 0.827 | 0.497 | 0.681 | 0.386 |
| 8 | Gibberellic acid | 0.02 | 24 | 0.746 | 0.698 | 0.503 | 0.615 | 0.833 |
| 9 | Gibberellic acid | 0.2 | 10 | 0.791 | 0.764 | 0.568 | 0.708 | 0.746 |
| 10 | Salicylic acid | 0.2 | 16 | 0.811 | 0.791 | 0.598 | 0.721 | 0.746 |
| 11 | Abscisic acid | 0.02 | 10 | 0.403 | 0.727 | 0.578 | 0.816 | 0.0621 |
| 12 | Abscisic acid | 0.02 | 14 | 0.682 | 0.84 | 0.559 | 0.85 | 0.246 |
| 13 | Abscisic acid | 0.02 | 6 | 0.28 | 0.643 | 0.598 | 0.736 | -0.08 |
| 14 | Abscisic acid | 0.2 | 8 | 0.348 | 0.663 | 0.586 | 0.78 | -0.029 |
| 15 | Abscisic acid | 0.2 | 10 | 0.561 | 0.737 | 0.577 | 0.815 | 0.046 |
| 16 | Abscisic acid | 0.2 | 12 | 0.551 | 0.81 | 0.567 | 0.838 | 0.132 |
| 17 | Abscisic acid | 2 | 10 | 0.48 | 0.686 | 0.561 | 0.801 | -0.188 |
| 18 | Abscisic acid | 2 | 14 | 0.521 | 0.733 | 0.542 | 0.835 | -0.05 |
| 19 | Abscisic acid | 20 | 10 | 0.51 | 0.497 | 0.407 | 0.577 | -0.12 |

**Supplementary Table 10. Test results of fucoxanthin prediction by ML models trained with raw data**

| **Model** | **MSE** | **RMSE** | **MAE** | ***R*^2^** |
| --- | --- | --- | --- | --- |
| Random Forest | 0.49 | 0.7 | 0.361 | 0.845 |
| Linear Regression | 1.27 | 1.127 | 0.92 | 0.598 |
| Support Vector Machine | 1.398 | 1.182 | 0.827 | 0.557 |
| Artificial Neural Network | 0.519 | 0.72 | 0.378 | 0.836 |

**Supplementary Table 11. Prediction of fucoxanthin yield using raw data**

| **S. No.** | **Name of the phytohormone** | **Concentration (mg l^-1^)** | **Days** | **Growth rate** | **Actual measured fucoxanthin yield (μg ml^-1^)** | **Predicted fucoxanthin yield by ML models** | | | |
| --- | --- | --- | --- | --- | --- | --- | --- | --- | --- |
|  |  |  |  |  |  | **RF** | **LR** | **SVM** | **ANN** |
| 1 | Control | 0 | 16 | 0.73 | 2.699 | 3.216 | 2.745 | 2.944 | 4.484 |
| 2 | Methyl jasmonate | 0.02 | 8 | 0.678 | 0.933 | 1.457 | 2.26 | 0.836 | 1.0367 |
| 3 | Indole acetic acid | 2 | 10 | 0.757 | 3.769 | 3.151 | 2.554 | 1.46 | 2.808 |
| 4 | Indole acetic acid | 2 | 18 | 0.969 | 4.276 | 5.023 | 3.763 | 3.433 | 6.107 |
| 5 | Gibberellic acid | 0.2 | 14 | 0.899 | 4.923 | 4.799 | 3.349 | 2.419 | 4.804 |
| 6 | Salicylic acid | 0.02 | 12 | 0.717 | 2.633 | 2.75 | 1.958 | 1.241 | 2.426 |
| 7 | Methyl jasmonate | 0.2 | 14 | 0.963 | 5.35 | 4.902 | 3.888 | 3.289 | 5.778 |
| 8 | Gibberellic acid | 0.02 | 24 | 0.746 | 0.107 | 0.902 | 2.472 | 1.036 | 1.418 |
| 9 | Gibberellic acid | 0.2 | 10 | 0.791 | 3.413 | 3.684 | 2.733 | 1.47 | 3.118 |
| 10 | Salicylic acid | 0.2 | 16 | 0.811 | 4.82 | 4.339 | 2.494 | 2.084 | 3.859 |
| 11 | Abscisic acid | 0.02 | 10 | 0.725 | 0 | 3.176 | 2.452 | 1.056 | 1.592 |
| 12 | Abscisic acid | 0.02 | 14 | 0.624 | 0.4 | 0.604 | 1.873 | 0.385 | 0.194 |
| 13 | Abscisic acid | 0.02 | 6 | 0.649 | 0 | 0.853 | 2.021 | 0.514 | 0.486 |
| 14 | Abscisic acid | 0.2 | 8 | 0.667 | 0 | 1.16 | 2.124 | 0.643 | 0.681 |
| 15 | Abscisic acid | 0.2 | 10 | 0.709 | 0 | 2.242 | 2.363 | 0.94 | 1.308 |
| 16 | Abscisic acid | 0.2 | 12 | 0.641 | 0.6 | 0.624 | 1.972 | 0.486 | 0.187 |
| 17 | Abscisic acid | 2 | 10 | 0.689 | 0 | 1.291 | 2.257 | 0.797 | 0.949 |
| 18 | Abscisic acid | 2 | 14 | 0.463 | 0 | 0 | 0.964 | -0.397 | -0.015 |


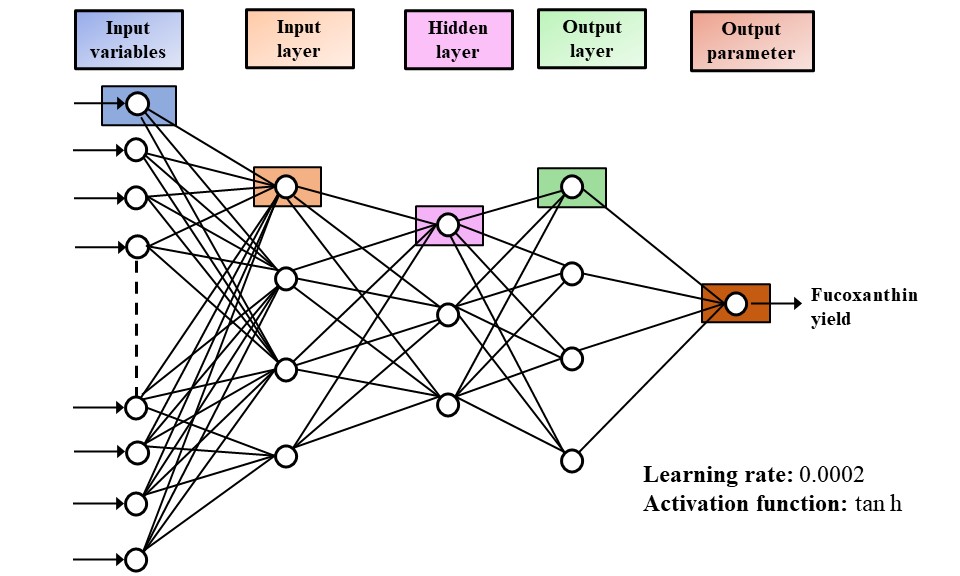


**Supplementary Figure 1.** Schematic representation of Artificial neural network ML model
